# Compound heterozygous variants in the multiple PDZ domain protein (MPDZ) cause a case of mild non-progressive communicating hydrocephalus

**DOI:** 10.1186/s12881-018-0540-x

**Published:** 2018-03-02

**Authors:** Nesreen K. Al-Jezawi, Aisha M. Al-Shamsi, Jehan Suleiman, Salma Ben-Salem, Anne John, Ranjit Vijayan, Bassam R. Ali, Lihadh Al-Gazali

**Affiliations:** 1 Department of Pathology, College of Medicine and Heath Sciences, United Arab Emirates University, P.O. Box 17666, Al-Ain, United Arab Emirates; 2 Department of Paediatrics, Tawam Hospital, Al-Ain, United Arab Emirates; 3 Department of Paediatrics, College of Medicine and Heath Sciences, United Arab Emirates University, P.O. Box 17666, Al-Ain, United Arab Emirates; 4 Department of Biology, College of Science, United Arab Emirates University, Al Ain, United Arab Emirates

**Keywords:** Congenital hydrocephalus, Multiple PDZ, *L1CAM*, Compound heterozygous variants, Autosomal recessive

## Abstract

**Background:**

Congenital hydrocephalus (CH) results from the accumulation of excessive amounts of cerebrospinal fluid (CSF) in the brain, often leading to severe neurological impairments. However, the adverse effects of CH can be reduced if the condition is detected and treated early. Earlier reports demonstrated that some CH cases are caused by mutations in *L1CAM* gene encoding the neural cell adhesion molecule L1. On the other hand, recent studies have implicated the multiple PDZ domain *(MPDZ)* gene in some severe forms of CH, inherited in an autosomal recessive pattern.

**Methods:**

In this study, whole-exome and Sanger sequencing were performed on a 9 months old Emirati child clinically diagnosed by CH. In addition, *in silico*, cellular, and molecular assays have been conducted to confirm pathogenicity of the identified variants and to establish disease mechanism.

**Results:**

Whole exome sequencing revealed two compound heterozygous novel variants (c.394G > A and c.1744C > G) in the affected child within the *MPDZ* gene. Segregation analysis revealed that each of the parents is heterozygous for one of the two variants and therefore passed that variant to their child. The outcome of the *in silico* and bioinformatics analyses came in line with the experimental data, suggesting that the two variants are most likely disease causing.

**Conclusions:**

The compound heterozygous variants identified in this study are the most likely cause of CH in the affected child. The study further confirms *MPDZ* as a gene underlying some CH cases.

## Background

Hydrocephalus is a serious and heterogeneous condition that is caused by an imbalance in the production and absorption of CSF. Acquired hydrocephalus develops as a result of environmental factors, whereas the congenital hydrocephalus, present at birth, is caused by either genetic reasons or development anomalies during fetal period [1, 2]. Until 1998, the estimated incidence of CH (excluding neural tube defects) was around 1.6 per 2000 live births. More recently, the estimation of prevalence of infantile hydrocephalus was estimated to be 1.1 per 1000 infants [3]. It is worth noting that the mortality rates of CH have declined to 66.3% in the United States over a 20-year study [4]. Several studies have suggested an autosomal recessive pattern of inheritance for some hydrocephalus cases without elucidating the causative genes [5–9]. In addition, it has been estimated that about 5% of CH cases are attributed to X-linked mutations in the *L1CAM* gene [10]. Additionally, mutations in *CCDC88C* gene encoding DAPLE protein (Dvl-Associating Protein With A High Frequency Of Leucine) were suggested to be the underlying cause of CH in three unrelated families from different ethnicities [1]. Another gene associated with hydrocephalus is the *AP1S2* which encodes a subunit of the adaptor protein 1 [11]. Interestingly, Al-Dosari et al., have recently reported cases from two Saudi families with congenital hydrocephalus and identified a founder homozygous mutation in *MPDZ* gene leading to a truncated protein. The pattern of disease in these families and segregation analysis clearly suggested a recessive mode of inheritance. The hydrocephalus in the two families was very severe leading to stillbirths in two of the affected cases, and death of several affected children in early infancy [12]. More recently, three novel null mutations were described in *MPDZ* gene in three unrelated hydrocephalic fetuses with multiple ependymal malformations. Histological and confocal studies performed on post-mortem sections revealed multifocal ependymal rosette formation and absence of MPDZ in ependymal lining [13].

MPDZ is an acronym from Multiple PSD95, DLG and ZO-1, they are protein modules found in many cytoplasmic proteins [14]. The *MPDZ* gene is located on the cytogenetic band 9p23 of chromosome 9, and encodes a 13-domain MPDZ protein that localizes at cellular junctions. In addition, numerous interactions have been documented for MPDZ suggesting that it might have multifaceted roles in the assembly and localization of the integral membrane proteins, including cell-cell junction. Hence, it regulates conductive permeability, prevents spontaneous acrosomal exocytosis and maintains GABA receptors, olfactory and melatonin receptor signaling [15]. Therefore, it is not surprising that various MPDZ domains bind to those receptors [16]. Moreover, MPDZ proteins are made of multiple domains that can bind multivalent scaffold proteins, and regulate intracellular signaling, receptor clustering and polarity of epithelial cells [17].

We report in this manuscript an Emirati family with a child affected by congenital non-progressive communicating hydrocephalus, who was found to be compound heterozygous for two novel likely pathogenic variants in the *MPDZ* gene. The phenotype of the affected child is milder than the previously reported cases associated with mutations in *MPDZ;* which indicates that the associated clinical phenotypes can be variable. This variability could be due to the nature of the variants and/or the presence of as yet unidentified disease-modifier genes.

## Methods

The participating family was referred to Tawam Hospital in Al Ain city (UAE) for evaluation of macrocephaly and communicating hydrocephalus in their first born male child aged 9 months. DNA samples from peripheral blood were extracted for the affected child and his parents, using Flexigene DNA extraction kit (Qiagen. Gmbh, Germany) following the manufacturers’ instructions.

### Whole-exome sequencing and variant analysis and prioritization

Whole-exome sequencing for the child was performed as a service at the Medical Genetic Laboratories of Baylor College of Medicine (www.bcmgeneticlabs.org). The output data from Illumina HiSeq were converted to FastQ files by CASAVA 1.8 software, and mapped by BWA program, the variant calls were performed using Atlas-SNP and Atlas-indel developed in –house by BCM HGSC. The variants were interpreted according to ACMG guidelines [18] and patient phenotypes. The WES report included minor allele frequency (MAF) data for variants reported in the NHLBI GO Exome Sequencing Project (ESP) ESP5400 database. For example, “1/3738 1/7017” in AA/EA, respectively. That means for the variant listed, the minor allele frequency was observed one time, the major allele was observed 3738 times in (AA). The annotated SNPs were mapped to dbSNP v131, v132 and 1000 Genome. Moreover, candidate variants were cross compared with Ensembl database [19], as well as to the Exome variant server [20]. In order to determine the effect of the candidate variants at the protein level, prediction analyses using Mutation Taster, Polyphen2 and SIFT/Provean were as well performed [21–25].

### Sanger DNA sequencing

The segregation of the candidate variants was confirmed by Sanger DNA sequencing in all available family members. PCR Primers were designed using Primer3 software [26] . Moreover, we checked the novelty of the variants from 100 ethnically matched control samples. Sanger sequencing was performed using Big Dye Terminator Kit (Applied Biosystems, USA) on 3130xl Genetic Analyzer (Applied Biosystems, USA). Clustal Omega algorithm was applied to analyze sequencing results [27].

### RNA extraction and cDNA sequencing

Total RNA was extracted from whole blood using SV Total RNA Isolation system (Promega, USA), then reverse transcribed with random primers and GoScript reverse transcriptase enzyme according to the manufacturer’s instructions (Promega, USA). After cDNA was synthesized, primers were designed to hybridize the flanking region, which contained the variant (c.394G > A) of the cDNA using Primer3 software. That region was then amplified with GoTaq® Flexi DNA polymerase (Promega, USA). The presence of the variant was confirmed using Sanger sequencing by Big Dye Terminator Kit (Applied Biosystems, USA) on 3130xl Genetic Analyzer (Applied Biosystems, USA).

### Generation of the mutant cDNAs for mammalian expression

Site-directed mutagenesis (SDM) was performed on the MPDZ expression clone (EX-E1329-M10), Accession No.: NM_003829.4 from GeneCopoeia (http://genecopoeia.com). Both identified variants (c.394G > A; Gly132Ser) and (c.1744C > G; Leu582Val) were generated individually on the c-Myc-His-tagged ORF cDNA clones using *Pfu* Ultra High-Fidelity DNA polymerase (Stratagene). The following are the primers which were designed according to QuikChange Site-Directed Mutagenesis Protocol,

MPDZ-Gly132Ser-Forward: 5’CAAAAATATGGCCAG**A**TCGCCATGTAGAAG3`.

MPDZ-Gly132Ser-Reverse: 5’CTTCTACATGGCGAC**T**CTGGGCCATATTTTTG3`.

MPDZ-Leu582Val-Forward: 5’CATTTTATCCGATCTGTT**G**TACCAGAGGGTCCTGTTG3`.

MPDZ-Leu582Val-Reverse: 5’CAACAGGACCCTCTGGTA**C**AACAGATCGGATAAAATG3`.

Successful introduction of the desired mutations was confirmed by Sanger sequencing using the Big Dye Terminator Kit (Applied Biosystems, USA) on the 3130xl Genetic Analyzer (Applied Biosystems, USA).

### Cell culture, transfection and confocal microscopy

HeLa cells were cultured in HyClone™ High Glucose Modified Eagles medium (GE Healthcare Life Sciences), supplemented with 10% fetal bovine serum, 4 mM L-Glutamine, 4500 mg/L Glucose, Sodium Pyruvate, and 100 U/ml Penicillin/Streptomycin. HeLa cells were incubated in a 37C° and 5% CO2 humidified incubator. When cells reached 60–80% confluency, they were seeded on sterile cover slips in 24-well culture plates. After 24 h of incubation, cells were transfected with either wild-type or mutant plasmids, and each was co-transfected with GFP-H-Ras plasmids using FuGENE HD (Promega). The GFP-H-Ras was used as a plasma membrane marker. 0.5 μg of each plasmid was incubated with Gibco™ Opti-MEM™ I Reduced Serum Media (Opti-MEM), followed by (3:1) ratio of (FuGENE:DNA), respectively. After 24 h of incubation at 37C^o^, cells were washed three times with PBS, fixed with absolute methanol then blocked with 2% bovine serum albumin (BSA) (Sigma) for 1 hour. Finally, cells were stained with mouse-anti-Myc-tag monoclonal antibody (dilution: 1:200; Cell Signaling Technology) for 1 hour. Followed by three times wash with PBS and incubation with anti-mouse IgG Fab2 Alexa Flour 555 antibody (dilution: 1:200; Cell Signaling Technology). Images were acquired using Nikon confocal Eclipse 80I microscope (Japan).

### Generation of the minigene construct, mutagenesis and transfection

The splicing cassette (p(13,17)/CMV) was designed by Deguillies and colleagues, which contained exons 13 and 17 with their downstream and upstream flanking intron of *4.1R* gene [28]. To demonstrate the splice-effect of the identified variant (c.394G > A) on the mRNA level, exon 5 and parts of its flanking introns were sub-cloned into the mini-cassette vector using BSTEII (**GGTCACC)** and NheI (**GCTAGC)** restriction enzymes (New England Biolabs), they were included in the following cloning primers: Forward primer: 5’-ATGTG**GGTCACC**ACACTTACTAAG-3′, Reverse primer: 5′- TATTATGGGTCATT**GCTAGC**ATGG-3′. After amplifying the desired genomic fragment of *MPDZ* gene which was around 647 bps, it was further purified using MinElute PCR Purification Kit (Qiagen. Gmbh, Germany). The respective enzymes were applied to digest the insert as well as the vector, and the latter was run along with its non-digested counterpart on gel electrophoresis in order to verify digestion. The digested vector was then gel-purified using MinElute Gel Extraction Kit (Qiagen. Gmbh, Germany). The PCR product of *MPDZ* gene fragment was then ligated to the vector and transformed. The clones were then sequenced to verify correct insertion.

To generate the variant in the minigene construct, site-directed mutagenesis (SDM) was performed on the vector harboring the wild-type *MPDZ* using the following primers: Forward primer: 5’-TCCTAAACAG**A**GTCGCCATG- 3′, Reverse primer: 5’-CATGGCGAC**T**CTGTTTAGGA-3′, the mutant vector was sequenced to confirm mutagenesis.

HEK 293 T cells were maintained in HyClone™ High Glucose Modified Eagles medium (GE Healthcare Life Sciences), supplemented with 10% fetal bovine serum, 4 mM L-Glutamine, 4500 mg/L Glucose, Sodium Pyruvate, and 100 U/ml Penicillin/Streptomycin. They were then seeded in a 6-well plate. On the following day, two wells were transfected with the wild-type minigene construct, while the two other wells were transfected with the mutant, and cultured for 48 h at 37 °C, 5% CO_2_. Cells were, then scraped and washed with PBS, and RNA was extracted using SV Total RNA Isolation system (Promega, USA), then reverse transcribed with random primers and GoScript reverse transcriptase enzyme according to the manufacturer’s instructions (Promega, USA). Subsequently, the resulting cDNA was analyzed on agarose gel electrophoresis, and sequenced to assess RNA splicing.

## Results

### Clinical report

**Fig. 1:**
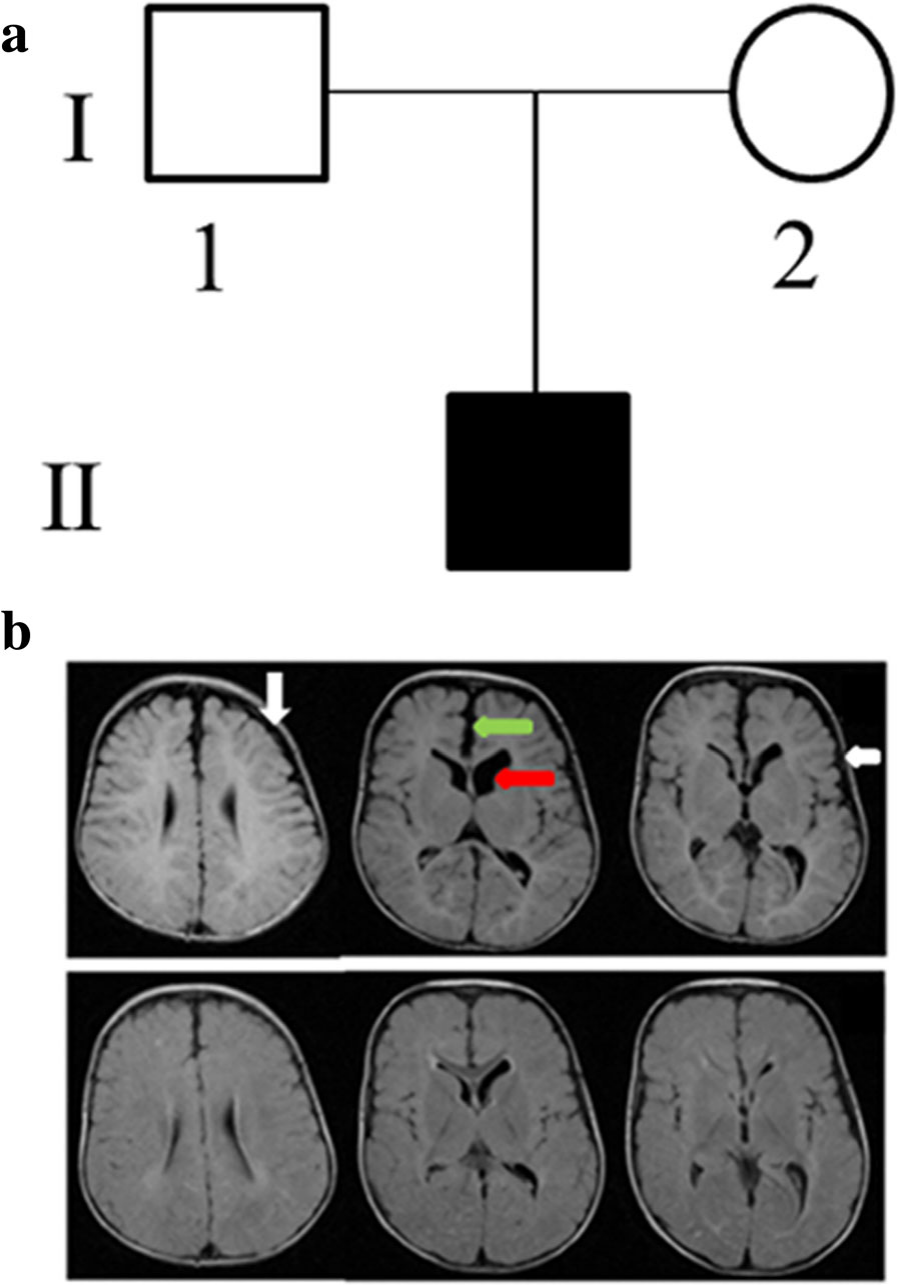
Clinical and molecular analysis of an Emirati family with a child affected by CH. **a** Pedigree showing the affected child with hydrocephalus for a non-consanguineous Emirati family. **b** MRI (T1 weighted images), upper panels at age 3 months showing widened CSF spaces in the frontoparietal regions (white arrows), widened interhemispheric fissure (green arrow) and mild dilatation of ventricles (red arrow). The lower panels at age of 17 months showing improvement in the above mentioned findings

The affected child was the first born son to a non-consanguineous couple from the UAE (Fig. 1a). The pregnancy was uncomplicated, but ultrasound at 20 weeks gestation revealed large head with dilated ventricles. Delivery was normal at term with a birth weight of 3400 g, length of 56 cm, and head circumference of 39 cm (>97th centile). There were no other reported neonatal problems. Family history revealed that the brother of the mother has a daughter with hydrocephalus and ventriculoperitoneal (VP) shunt. Unfortunately, no further clinical or radiological information were available for this child because the family refused to participate in the research; and hence, no clinical or molecular studies were performed on this branch of the family.

MRI brain at 3 months revealed widened CSF spaces in the frontoparietal region bilaterally, as well as mild dilatation of lateral and third ventricles (Fig. 1b). The cerebral volume was preserved and the white matter appeared normal. Findings were thought to be consistent with external hydrocephalus [29].

The patient was evaluated in our Genetics Clinic at the age of 9 months. His weight was 10 kg (75^th^ centile), length 77 cm (90^th^–95^th^ centile), and head circumference 50 cm (> 97^th^ centile; Z score 3.34). He had large head with frontal bossing and high arched palate, but no other dysmorphic features. He had normal developmental milestones. At the age of 14 months he was walking and saying a few words, and continued to have a large head. At 15 months his weight was 11.5 kg (> 50^th^centile), height 84 cm (> 90^th^ centile) and head circumference 51 cm (> 97^th^ centile). Detailed ophthalmological examination was normal. Progress brain MRI at age 17 months showed less dilatation of ventricles and less widening of CSF spaces, in keeping with the natural progress of external hydrocephalus. Negative investigations included: microarray, urine organic acid, urine mucopolysaccharides (MPS), creatine phosphokinase (CPK) and skeletal survey.

### Whole-exome sequencing revealed that the affected child has two compound heterozygous variants in the *MPZD* gene

**Fig. 2:**
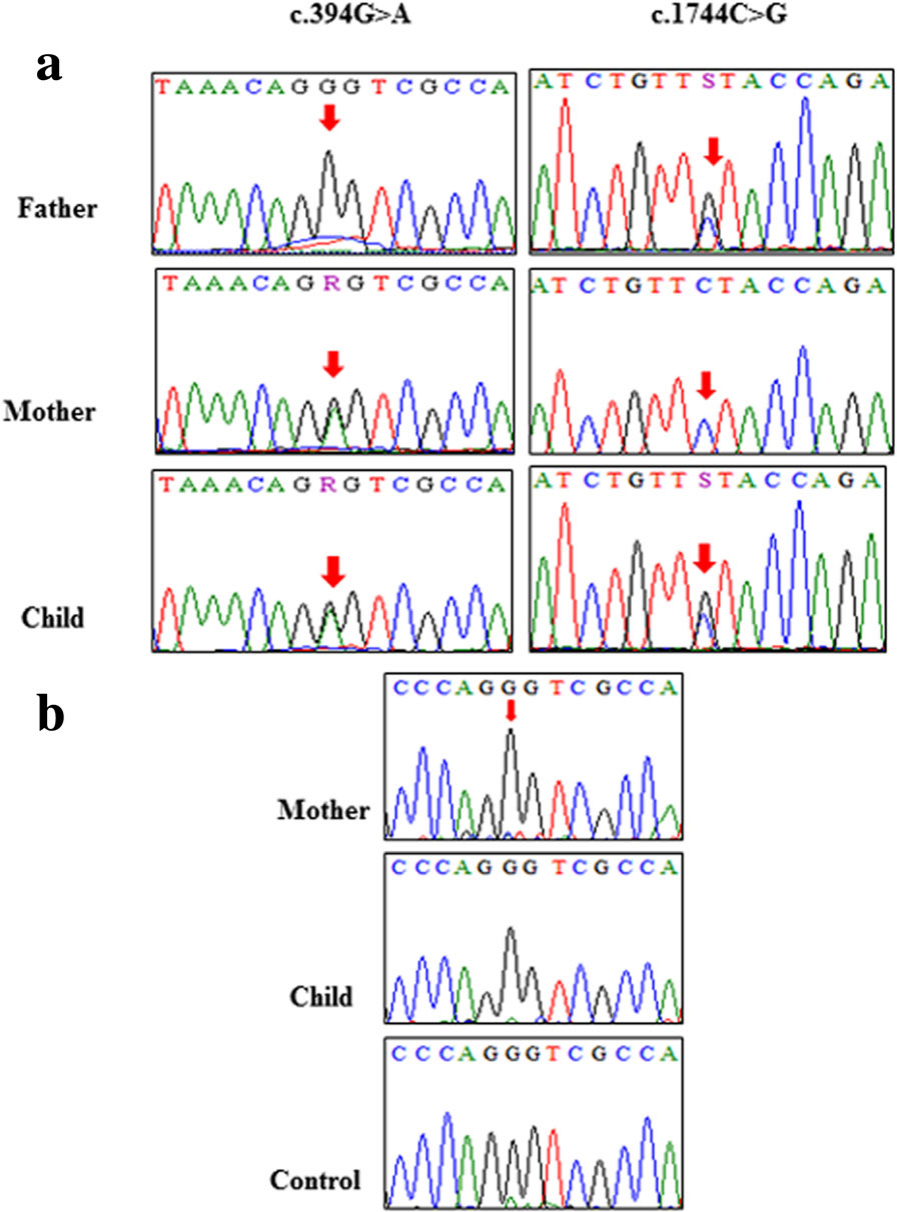
**a** Chromatograms of DNA sequence changes in the *MPDZ* gene in this family. A compound heterozygous variant in the child c.394G > A (left panels), inherited from his mother and a second c.1744C > G variant (right panels) inherited from his father. Parents are both carriers for each variant (Red arrows indicate the positions of the variants). **b** Chromatograms of cDNA from the mother, child and a healthy control, showing the wild nucleotide (G) at position c.394 of *MPDZ* gene (indicated by a red arrow)

Clinical whole-exome sequencing analysis at Baylor College Genetics Laboratory revealed two compound heterozygous variants (c.394G > A and c.1744C > G) in the *MPDZ* gene. Sanger sequencing confirmed the segregation of these compound heterozygous variants in this family with the child being compound heterozygous for both variants (Fig. 2a). The mother was heterozygous for the c.394G > A variant (Fig. 2a, left panels), while the father was heterozygous for the c.1744C > G variant (Fig. 2a, right panels). The first variant (c.394G > A) has been reported as a SNP (rs201101621); which leads to the substitution of glycine at 132 with serine (p.Gly132Ser). However, this SNP has a minor allele frequency of T = 0.0004 based on 1000 genome, and no homozygote were reported for this variation. In addition, computational analysis predicted that this variant is disease causing/damaging using Mutation Taster and SIFT/Provean software. More importantly, this change was also predicted to cause acceptor splice site change by Neural Network software (www.fruitfly.org) [30]. It predicted a score of 0.78 for the acceptor splice-site present at c.394-1G, which is one nucleotide upstream to the variant. Furthermore, the software predicted that the variant might lead to the use of the cryptic acceptor-splice sites within intron 5–6 with a score of 0.74, leading to skipping of exon 5 and the generation of a stop codon.

The second variant (c.1744C > G) was predicted to cause a missense mutation at amino acid position leucine 582 (p.Leu582Val). At the same locus, a different SNP (rs201275925) has been reported with G/A alleles. This SNP had a minor allele frequency of A = 0.0002. However, the change was synonymous. *In silico* analysis using Mutation Taster predicted that the p.Leu582Val variant was disease causing. However, SIFT/Provean predicted that the change was tolerable/neutral. Both variants were absent in Exome Variant Server for all the tested population. Furthermore, there were no damaging variants in *MPDZ* gene in a homozygous state in normal individuals in this database. Likewise, there were no reports of any homozygous damaging variations at the same locus in *MPDZ* gene in NCBI. Additionally, WES analysis data identified two variants in heterozygous states in genes known to be implicated in hydrocephalus. One of which was in *CCDC88C* gene (c.4327G > A) on chromosome 14; a previously reported reference SNP (rs189215037). This variant leads to an amino acid substitution (p.Ala1443Thr) which was predicted to be tolerated/benign by SIFT/PolyPhen-2 tools with ESP5400 AA/EA frequency of 0/3086 16/6636. The other variant was found in *KIF7* gene (c.2777G > A) on chromosome 15 which was predicted to be a tolerable amino acid substitution (p.Arg926Gln), with a frequency was not reported in ESP5400 AA/EA. In addition, heterozygous variants in these two genes are not predicted to be disease-causing and therefore were excluded as relevant to the phenotype in the child.

### cDNA sequencing revealed a likely unstable copy of the c.394G > A variant mRNA

It was predicted that c.394G > A variant would result in a splice site change. *MPDZ* cDNA from the mother, the affected child and from a healthy control were all prepared and sequenced. In all three cases, a single sequencing peak (G) at position c.394 was observed (Fig. 2b). This was different from what we observed from the genomic DNA sequencing of the affected child and mother (Fig. 2a (left panels). This discrepancy can be explained by the activation of nonsense-mediated decay (NMD) of the mutated copy of the mRNA, but not the wild type. This activation could be due to the introduction of the predicted premature stop codons.

### The c.394G > A variant caused exon 5 skipping

**Fig. 3:**
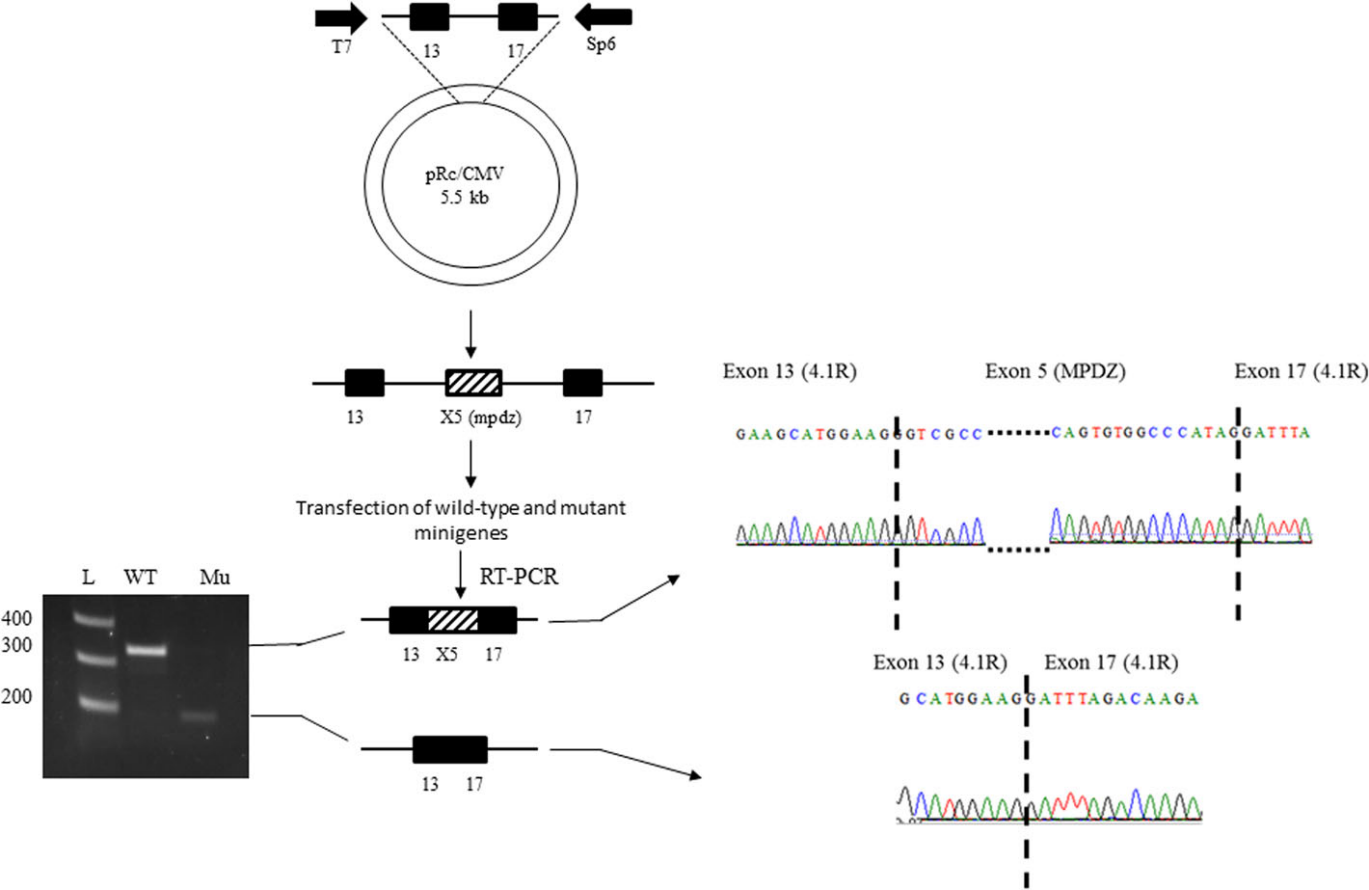
Illustrative model of the mini-gene analysis protocol. Patterned box depicts exon 5 and its flanking introns of the subcloned *MPDZ* gene, black ones depict exons 13 and 17 of *4.1R* gene which was subcloned into pRc/CMV vector. Splicing of the wild type cDNA shows the authentic splicing and the inclusion of the three exons and that is noticed from the size of the amplified band on the gel (WT). Whereas, the mutant cDNA (Mu) shows a smaller band due to cryptic splicing and exon skipping. On the right side, the chromatograms represent both splice variants, dashed lines separate the joined exons

We wanted to further investigate the effect of c.394G > A on mRNA splicing, therefore exon 5 and its flanking regions of *MPDZ* gene were sub-cloned into a mini-cassette vector used for the analysis of splicing (Fig. 3). RNAs extracted from the transfected cells harboring wild type and mutant mini-cassette vectors were converted into cDNAs and then analyzed by agarose gel electrophoresis which revealed an aberrant band with a molecular weight of less than 200 bps appeared compared to the wild type band of 300 bps (Fig. 3). Upon sequencing both bands, the mutant band revealed skipping of exon 5 of *MPDZ*, while conserving both of the vector’s exons, just as expected by the neural network prediction tool. On the other hand, the wild-type cDNA retained exon 5 along with the vector’s exons (Fig. 3).

### Protein modeling of the p.Leu582Val variant indicated possible disruption of the protein structure

**Fig. 4:**
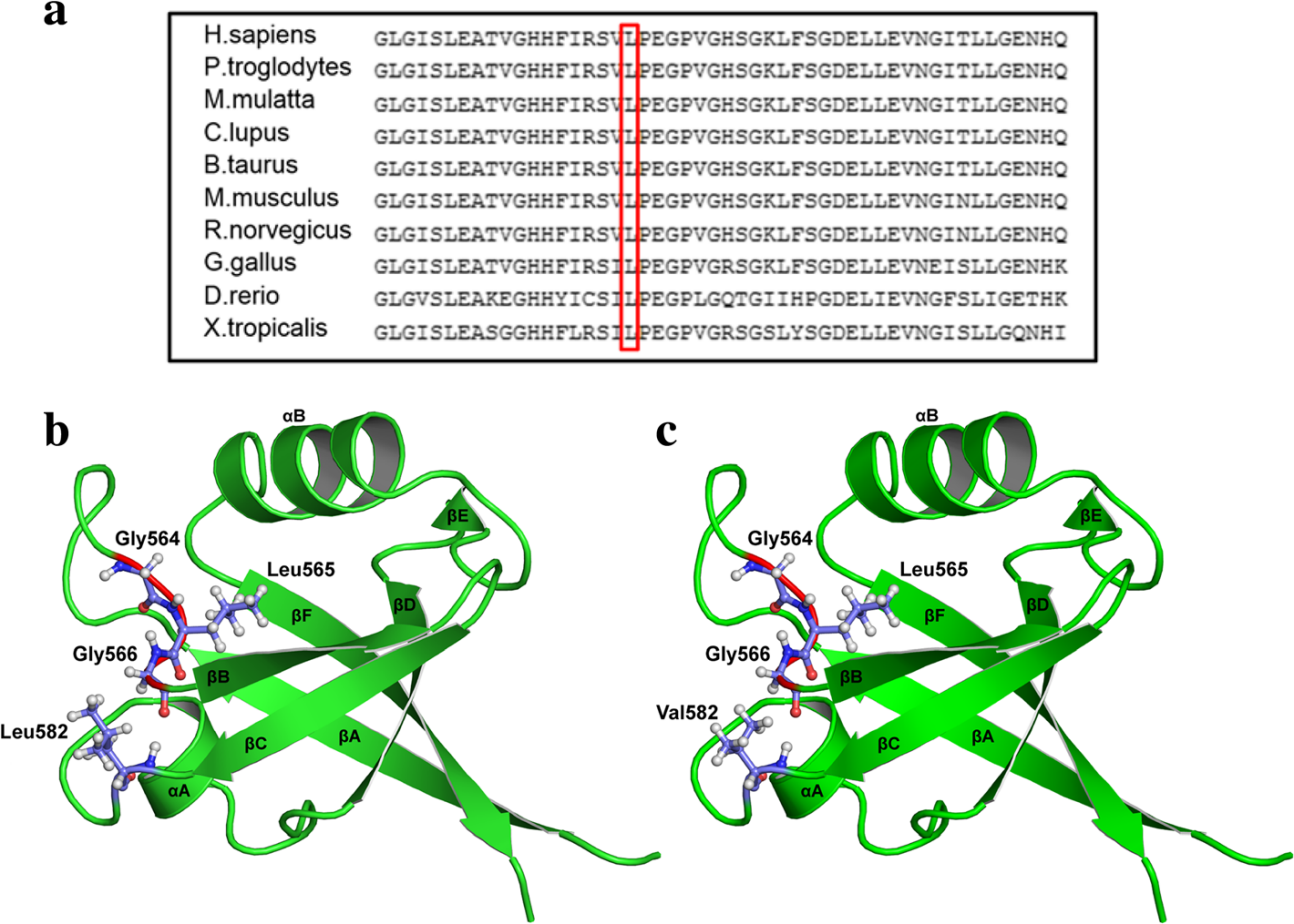
**a** Multiple sequence alignments for MPDZ protein among different Eukaryotic species by Clustal Omega show the conservation of amino acid leucine among selected Eukaryotic species. **b** The rat PDZ4 (wild type) was used to generate the image in PyMOL. The conserved GLG loop is shown in red with ball and stick representation. The secondary structures (βA, βB, βC, αA, βD, βE, αB, βF) are shown with the wild –type Leucine located between βC and αA **c** PDZ4 (mutant type). The rat PDZ4 structure (PDB ID: 4XH7) was used to generate a model of the human PDZ4 domain using Schrodinger Prime. The image was generated in PyMOL.. The mutant valine which lies between βC and αA could disrupt or interfere with the GLG loop

The second variant (c.1744C > G) affected a highly conserved residue **(**p.Leu582Val) (Fig. 4a). The images in fig. 4b and c were generated using Schrödinger Prime and rendered using PyMOL. The variant which was located within the MPDZ protein was predicted to cause functional disruption of the domain. The mutant amino acid valine lies between βC and αA of PDZ4, interfering with the conserved GLG repeat which is essential for hydrogen bond coordination of the c-terminal carboxylate group. Moreover, as already mentioned, the site of leucine at this position displays evolutionary conservation consistent with the damaging nature of the change at that position.

### Confocal analysis revealed that the pathogenicity of the compound heterozygous variants is not due to subcellular localization defects

**Fig. 5:**
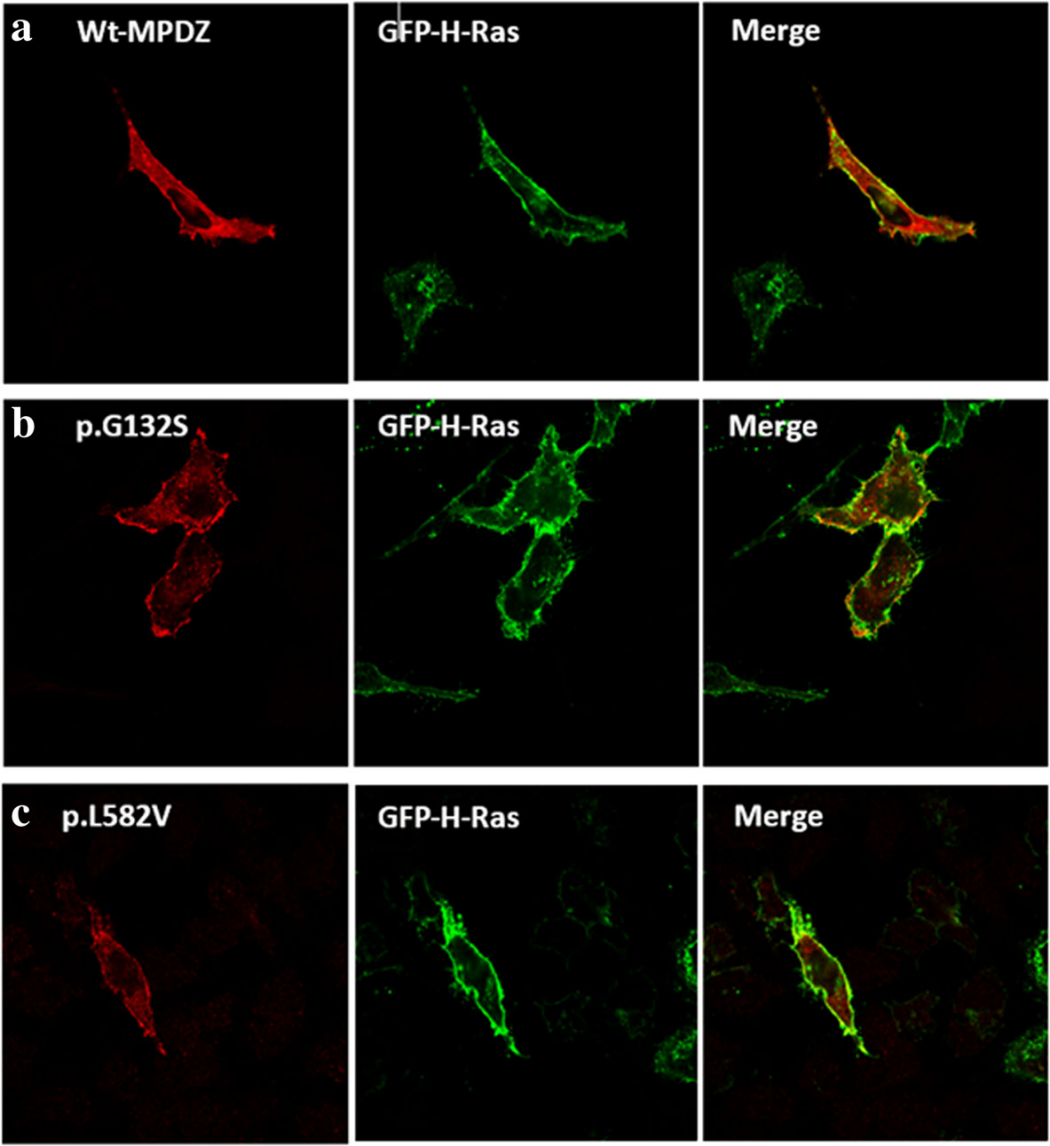
A comparison of the intracellular localization of the wild type MPDZ protein and both mutants. **a** Myc-tagged wild-type (Wt) MPDZ protein. **b** Myc-tagged (p.Gly132Ser) MPDZ protein. **c** Myc-tagged (p.Leu582Val) MPDZ protein. They were immunostained with anti Myc antibodies (red). Cells were co-transfected with GFP-H-Ras plasmid (green) which localizes at cell membrane. Images reveal that the wild-type protein, as well as both mutant proteins (red) co-localize largely and similarly with GFP-H-Ras at the plasma membrane, in addition to the localization of MPDZ in the cytosol

To establish whether the pathogenicity of the amino acid substitution (p.Leu582Val) is due to the subcellular mislocalization of the mutant protein, HeLa cells were transfected with plasmids harboring the wild type and the mutant MPDZ cDNAs. Although we have shown that the c.394G > A variant is altering RNA splicing, we wanted to test the alternative possible implication of this variant (i.e. generating of a missense mutation at position p.Gly132Ser), we therefore generated this change as well, using the MPDZ cDNA and then expressed the mutants (p.Leu582Val, p.Gly132Ser) and wild type in HeLa cells. In all cases, cells were co-transfected with GFP-H-Ras as a plasma membrane marker. MPDZ protein is known to co-localize with synaptic markers and with tight junctions such as claudin-5 at cell membranes [31], as well as the cytosol [32]. Confocal imaging showed that the localization of the two variants is similar to that of the wild type (Fig. 5), thus, excluding the possibility that the pathogenicity of both variants is linked to mis-localization of the MPDZ protein.

## Discussion

The reported child has congenital communicating hydrocephalus with no associated abnormalities and near normal development. The hydrocephalus was non-progressive and did not require shunting. The affected child was found to have compound heterozygous variants in the *MPDZ* gene. A single homozygous CH-causing mutation in this gene has been recently reported in two individuals from two unrelated Saudi Arabian families. The phenotype in these previously reported families is much more severe compared to our patient. In addition to a severe progressive hydrocephalus requiring shunting, other abnormalities were reported in the affected individuals including simplified gyration pattern in all of them, and coloboma with congenital heart disease in one of them. In addition, there were two still births presumably due to CH in one family and six CH cases in the second extended family. The cognitive outcome of the two long term survivors in Al-Dosari et al. (2013) correlated with the speed of neurosurgical intervention initiation after birth [12]. Moreover, five hydrocephalic stillbirths from three unrelated families were identified. Three probands were genetically tested to find null mutations in *MPDZ* gene. Medical termination was achieved to all fetuses except for one in whom the child died from severe hydrocephalus [13]. Table 1 lists all the previously reported mutations in *MPDZ* gene in patients with congenital hydrocephalus.

**Table 1:** Reported *MPDZ* mutations in patients with congenital hydrocephalus

DNA Change	Protein Change	No. of Patients	Origin	Phenotypes	Ref.
c.628C > T	p.(Gln210*)	1	Saudi	Severe congenital supratentorial hydrocephalus, mild hypotonia, coloboma, atrial septal defect	[12]
c.628C > T	p.(Gln210*)	1	Saudi	Severe congenital supratentorial hydrocephalus	[12]
c.1291_1294del	p.(Val431Metfs*14)	2 sibs	Senegalese	Massive hydrocephalus	[13]
c.533 + 1G > T	Frame-shift	2	–	Foetal macrosomia with severe isolated ventriculomegaly	[13]
c.2248C > T	p.(Arg750*)	3 sibs	–	Recurrent hydrocephalus	[13]
(Compound heterozygous) c.394G > A, c.1744C > G	Predicted: p.Gly132Ser Experimentally shown to cause exon 5 skipping p.Leu582Val	1	Emirati	Congenital non-progressive communicating hydrocephalus with normal developmental milestones	Current study

**Fig. 6:**
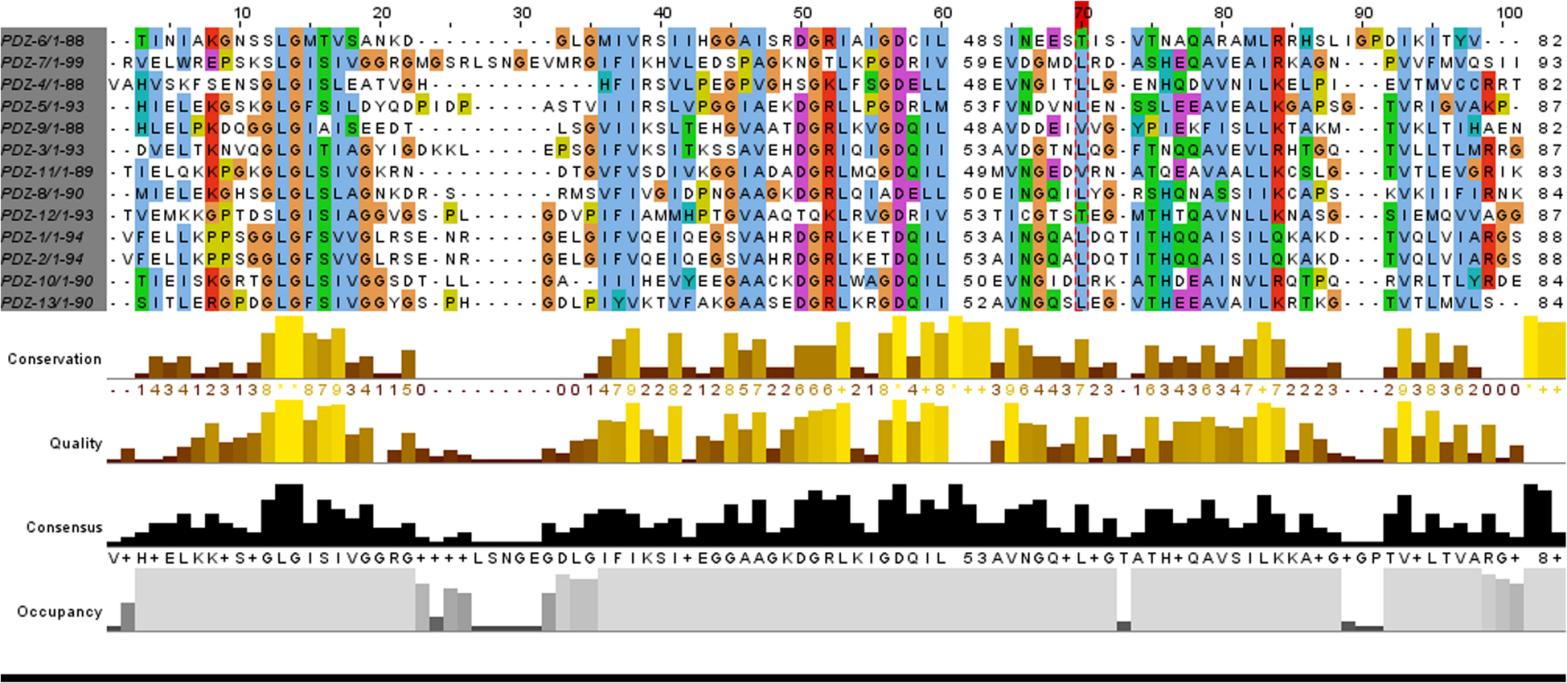
The alignment of the 13 PDZ domains in MPDZ protein of human, indicating the homologous regions in colored boxes showing a graphical view of those conserved regions by Jalview [40]

The mutation reported by Al-Dosari et al. (2013), led to a 12-domain truncated protein out of, originally 13 domains, abolishing most of the interactions with other proteins and rendering the affected members severely hydrocephalic. This mutation likely leads to a complete loss-of-function of the MPDZ protein. Compared to our study, we detected a splice site and missense variants that might, provisionally affect domains (between L27 and PDZ-1, as well as PDZ4, respectively). MPDZ protein has many possibilities of interactions due to its multiple-domain structure. Each domain is highly conserved among species and each is made up of six beta sheets and two alpha helices (Fig. 4b and c). Furthermore, the 13 PDZ domains are > 50% homologous in rat MUPP1/MPDZ protein. [33] We present in fig. 6 the alignment of the 13 domains in human MPDZ, showing homology in the preserved GLG loop, as well as other locations. This loop lies between βA and βB sheets. It functions as a coordinator for hydrogen bond of the C-terminal carboxylate group of the binder protein. Another important domain in MPDZ is the hydrophobic pocket, which lies between βB and αB, constituting the canonical binding sites for MPDZ. Opposite to this binding groove, at about 30 residues C-terminal extension of the PDZ domain lie other non-canonical binding sites [34]. In the present study, the change from leucine (non-polar and bulkier side chain) to valine (non-polar with smaller side chain) at position 582 is predicted to be disturbing the GLG loop (Fig. 4c). Despite the fact that glycine is neither at the GLG loop nor within the hydrophobic pocket, it is predicted that the reported variant c.394G > A is in an acceptor splice site region, leading to a stop codon and truncation of the majority of the protein domains, rendering the protein with only L27 domain. PDZ-1 domain enhances binding of other PDZ domains. For example, PDZ-2 domain of Syntenin (a PDZ domain that functions as an adaptor) binds neurexin, ephrin-1 and syndecan only when paired with PDZ-1 [35, 36]. Moreover, Grootjans et al. (2000) showed that PATJ, a protein involved in cell polarity and a mammalian paralogue of MPDZ, has a PDZ-1 domain linked to L27 domain. When they decreased both domains in mammalian cell culture, it resulted in disassembly in tight junctions. Strikingly, they had to be linked together for PATJ to function properly [36].

Moreover, the absence of the mutated copy of mRNA that was revealed by sequencing the cDNA for the mother and child suggested an NMD mechanism which is consistent with the prediction of the variant (c.394G > A) affecting an acceptor splice-site according to Neural Network prediction tool. Following that, we had to investigate the effect of that variant on *MPDZ* expression by quantifying mRNA using real time PCR. Unfortunately, MPDZ is expressed at very low levels in the hematopoietic lineages (https://www.ncbi.nlm.nih.gov/unigene). However, transcripts could be detected in patient blood samples by RT-PCR, which enabled further characterization of the mutant. Besides, higher tissue sources expressing *MPDZ* such as skin fibroblasts could not be obtained from the patient.

Furthermore, to experimentally verify the effect of the variant (c.394G > A) on mRNA splicing and to reveal the possible disease mechanism; mutant and wild mini-gene constructs were designed to analyze splicing variants [37]. Sequencing both cDNAs revealed the actual adjoining of the exons in the wild-type, while skipping of exon 5 of *MPDZ* gene within the mutant. This is explained by the fact that the variant (c.394G > A) is located at the first nucleotide of exon 5which was predicted to cause a splice-site aberration (Fig. 3).

The second variant (c.1744C > G), leucine is also conserved at that position among multiple species (Fig. 4a). It lies between βC sheet and αA of PDZ-4 domain. This change could be disease causing/probably damaging according to Mutation Taster and PolyPhen, respectively. It has been shown that binding of PDZ-5 domain of GRIP (Glutamate receptor interacting protein) to its receptor GluR2 failed to occur in the absence of PDZ-4 domain, indicating its role in the proper folding of the other domain [38]. Although our variant did not take place in any of the canonical binding sites of MPDZ, function of PDZ-4 was not only restricted to such binding, rather PDZ-4 was shown to be involved in the direct binding of PDZK1 (a MPDZ domain-containing protein in hepatocytes which controls the expression and localization of HDL receptors) to the inner leaflet of the cell membrane. And that this binding was not due to any of the four PDZ domains in that protein, but to PDZ-4 solely [39]. It was assumed that since MPDZ localizes at cell-cell adhesion sites and is involved in regulating permeability, mutations in this protein would disrupt this localization. Hence, alteration in CSF secretion would result in hydrocephalus. After performing confocal microscopy on the wild type and mutant plasmids, we observed no difference in the subcellular localization of either mutant compared to the wild type. Therefore, we assume that the pathogenesis of these missense changes is not due to trafficking defects; rather it could be due to disruption or complete loss of the protein function or an essential protein-protein interaction.

## Conclusions

The identified compound heterozygous variants detected by whole-exome sequencing and confirmed by Sanger sequencing in the affected child are the most likely cause of his CH. The deleterious effects of the two variants have been illustrated experimentally and by bioinformatics tools. The clinical presentation is consistent with CH which we strongly argue to be caused by the compound heterozygous variants in *MPDZ* gene.

## Abbreviations

AA: African American
CH: Congenital hydrocephalus
CPK: Creatine phosphokinase
CSF: Cerebrospinal fluid
DAPLE: Dvl-Associating Protein With A High Frequency Of Leucine
EU: European
GRIP: Glutamate receptor interacting protein.
*L1CAM*: L1 Cell Adhesion Molecule
MAF: Minor Allele Frequency
MPDZ: Multiple PDZ
MPS: Mucopolysaccharides
NMD: Nonesense-mediated decay
SDM: Site-directed mutagenesis
VP: Ventriculoperitoneal
